# Supplementary material for: Interleukin-17 and Th17 Lymphocytes Directly Impair Motoneuron Survival of Wildtype and FUS-ALS Mutant Human iPSCs
Source: Int J Mol Sci. 2021 Jul 27;22(15):8042. doi: 10.3390/ijms22158042 (PMC8348495; doi:10.3390/ijms22158042)
Supplement: Supplementary file 1 [file ijms-22-08042-s001.zip › ijms-1281094-supplementary.pdf]

Supplementary Material

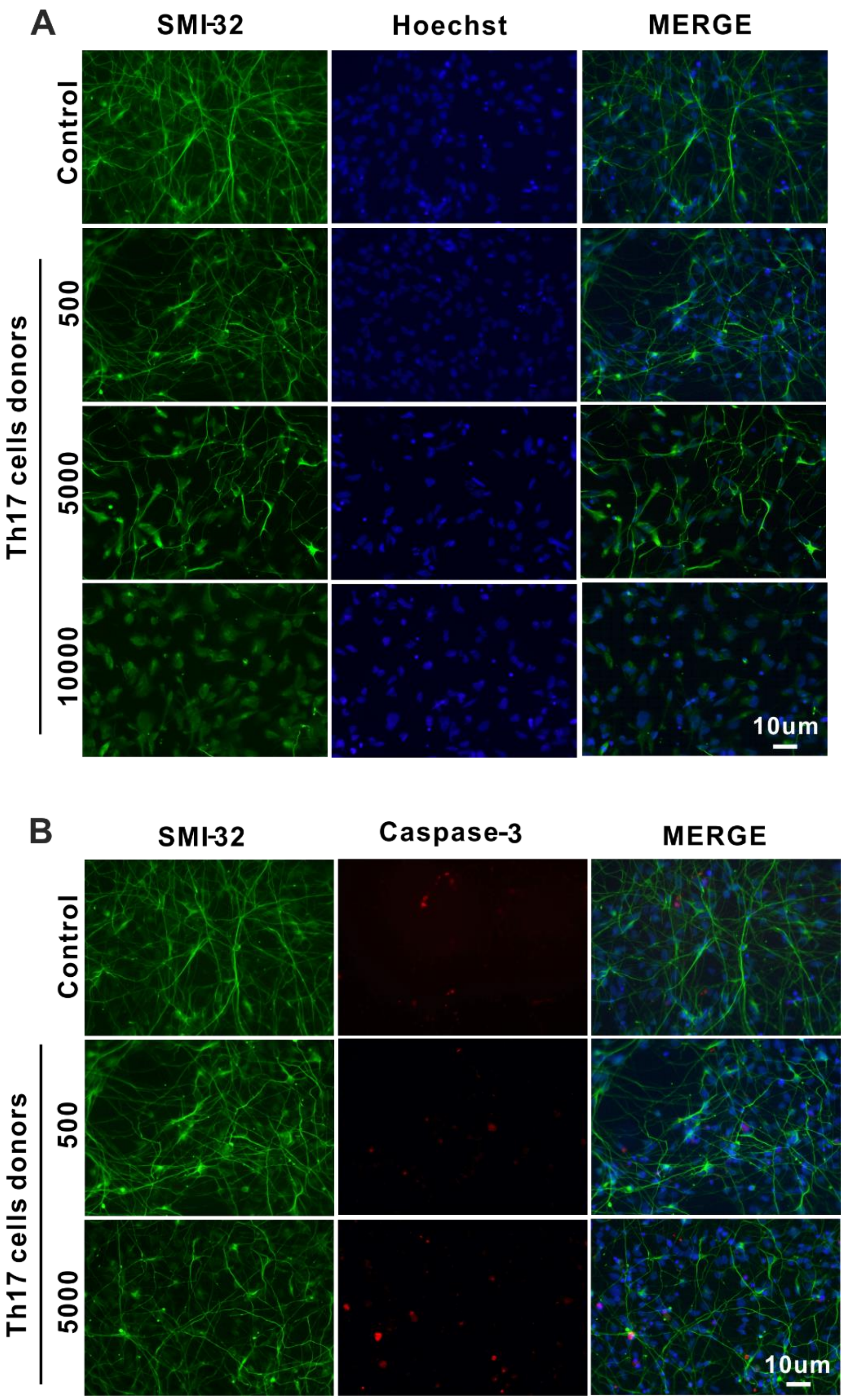

Figure S1. The effect of the different numbers of Th17 cells on WT MNs. (**A**) Immunostaining MNs with SIM-32 antibody in co-culture with Th17 cells (500, 5000, and 10,000) from MS for 24 h. (**B**) Immunostaining MNs with SIM-32 and caspase-3 antibody in co-culture with Th17 cells (500, 5000) from MS for 24 h.

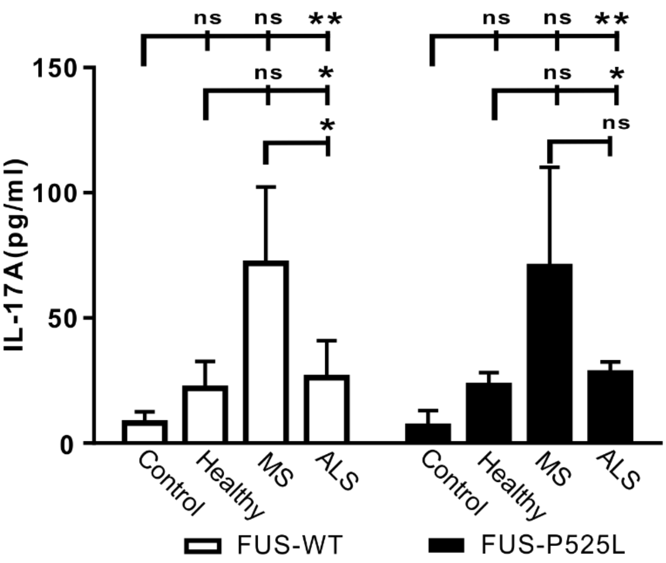

Figure S2. The cytokines in the supernatants of the co-culture model. Levels of IF-17A in the different co-culture mediums. *$P < 0.05$, **$P < 0.01$, versus controls. Ns—no significance

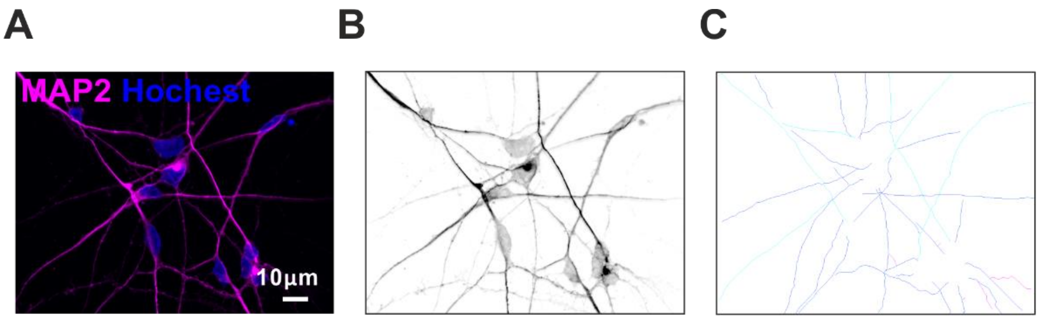

Figure S3. Representative image for neurite length analysis. (**A**) The image of how the neurite length was analyzed in this study. (**B**) The converted image was performed by image J. (**C**) The Snapeshot image of Neruon J tracings.

Table S1. Exemplarily, the summary of quantification of neurite length is depicted for the analysis within Figure 1D.

Name	MNs Numbers (*n*)	Mean ± SD (% of Control)
WT Control	110	106.7 ± 7.1
Healthy	101	80.4 ± 8.3
ALS	99	77.1 ± 6.4
MS	109	57.9 ± 1.9
FUS P525L Control	118	97.7 ± 7.8
Healthy	120	80.3 ± 2.5
ALS	105	80.0 ± 11.3
MS	107	55.3 ± 12.5
